# Supplementary material for: Hippo Pathway Dysregulation in Thymic Epithelial Tumors (TETs): Associations with Clinicopathological Features and Patients’ Prognosis
Source: Int J Mol Sci. 2025 Jun 20;26(13):5938. doi: 10.3390/ijms26135938 (PMC12250049; doi:10.3390/ijms26135938)
Supplement: Supplementary file 1 [file ijms-26-05938-s001.zip › S1 Antibody Datasheets.pdf]

For Research Use Only

LATS1 Polyclonal antibody

Catalog Number: 17049-1-AP Featured Product 38 Publications

proteintech®
Antibodies | ELISA kits | Proteins
www.ptglab.com

Basic Information

Catalog Number:
17049-1-AP

Size:
150ul , Concentration: 1000 μg/ml by Nanodrop;

Source:
Rabbit

Isotype:
IgG

Immunogen Catalog Number:
AG10709

GenBank Accession Number:
BC015665

GeneID (NCBI):
9113

Full Name:
LATS, large tumor suppressor, homolog 1 (Drosophila)

Calculated MW:
15 kDa, 127 kDa

Observed MW:
140-150 kDa, 120 kDa

Purification Method:
Antigen affinity purification

Recommended Dilutions:
WB 1:500-1:2000
IHC 1:50-1:500
IF 1:50-1:500

Applications

Tested Applications:
IF, IHC, WB, ELISA

Cited Applications:
IF, IHC, WB

Species Specificity:
human, mouse

Cited Species:
human, sheep, mouse

Note-IHC: suggested antigen retrieval with TE buffer pH 9.0; (*) Alternatively, antigen retrieval may be performed with citrate buffer pH 6.0

Positive Controls:

WB : HepG2 cells, K-562 cells, SH-SY5Y cells, HT-29 cells, A549 cells, C2C12 cells

IHC : human breast cancer tissue, human testis tissue

IF : HeLa cells,

Background Information

LATS1(Large tumor suppressor homolog 1) is also named as WARTS and belongs to the AGC Ser/Thr protein kinase family. The gene encodes a highly conserved (from fly to human) protein kinase that plays a crucial role in the prevention of tumor formation by controlling the progression of mitosis. The expression of both long(170 kDa) and short lats1 isoforms(120 kDa) in vertebrate retinal cells raises the possibility that these lats1 proteins may act as negative key regulators of the cell cycle each of them performing a unique role (PMID:15777619). In mammalian cells, LATS1 was phosphorylated in a cell cycle-dependent manner and complexed with CDC2 in early mitosis (PMID:9988268). LATS1 also can be detected as 120 kDa and 140-150 kDa, and play a key role in the regulation of Hippo pathway (PMID: 27940445).

Notable Publications

Author	Pubmed ID	Journal	Application
Zakiyatul Faizah	32987643	Molecules	WB
Deqiang Kong	34508829	Biochim Biophys Acta Mol Basis Dis	WB
Xiehong Liu	30256412	J Cell Physiol	WB

Storage

Storage:
Store at -20°C. Stable for one year after shipment.
Storage Buffer:
PBS with 0.02% sodium azide and 50% glycerol pH 7.3.
Aliquoting is unnecessary for -20°C storage

*** 20ul sizes contain 0.1% BSA

Selected Validation Data

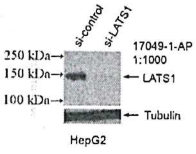

WB result of LATS1 antibody (17049-1-AP; 1:1000; incubated at room temperature for 1.5 hours) with sh-Control and sh-LATS1 transfected HepG2 cells.

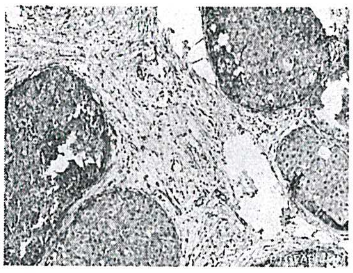

Immunohistochemical analysis of paraffin-embedded human breast cancer tissue slide using 17049-1-AP (LATS1 antibody) at dilution of 1:200 (under 10x lens). Heat mediated antigen retrieval with Tris-EDTA buffer (pH 9.0).

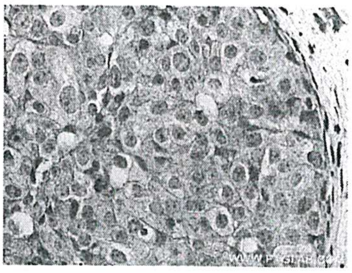

Immunohistochemical analysis of paraffin-embedded human breast cancer tissue slide using 17049-1-AP (LATS1 antibody) at dilution of 1:200 (under 40x lens). Heat mediated antigen retrieval with Tris-EDTA buffer (pH 9.0).

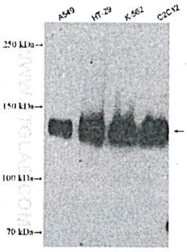

Various lysates were subjected to SDS PAGE followed by western blot with 17049-1-AP (LATS1 antibody) at dilution of 1:6000 incubated at room temperature for 1.5 hours.

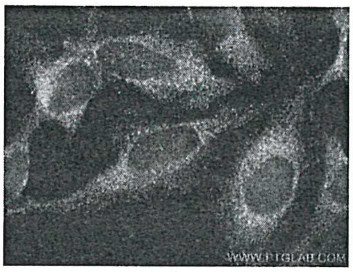

Immunofluorescent analysis of (-20°C Methanol) fixed HeLa cells using LATS1 antibody (17049-1-AP) at dilution of 1:200 and CoraLite®488-Conjugated AffiniPure Goat Anti-Rabbit IgG(H+L).

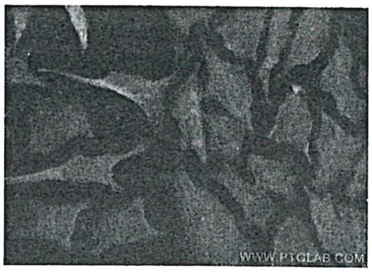

Immunofluorescent analysis of (-20°C Methanol) fixed HeLa cells using LATS1 antibody (17049-1-AP) at dilution of 1:200 and CoraLite®488-Conjugated AffiniPure Goat Anti-Rabbit IgG(H+L).

invitrogen

Catalog # PA5-98902

MOB1 Polyclonal Antibody

Product Details

Size	100 µg
Species Reactivity	Human
Host/Isotype	Rabbit / IgG
Class	Polyclonal
Type	Antibody
Conjugate	Unconjugated
Immunogen	Recombinant Human MOB kinase activator 1A protein (2-216AA)
Form	Liquid
Concentration	5 mg/mL
Purification	Protein G
Storage buffer	PBS, pH 7.4, with 50% glycerol
Contains	0.03% ProClin 300
Storage conditions	-20°C or -80°C if preferred
RRID	AB_2813515

Applications	Tested Dilution	Publications
Western Blot (WB)	1:2,000-1:5,000	-
Immunohistochemistry (Paraffin) (IHC (P))	1:100-1:1,000	-
Immunocytochemistry (ICC/IF)	1:200-1:500	-
ELISA (ELISA)	Assay-Dependent	-
Immunoprecipitation (IP)	1:200-1:2,000	-

abcam

ab205270

Anti-active YAP1 antibody [EPR19812]

Rabbit Recombinant Monoclonal YAP1 antibody. Validated in WB, ICC/IF, IHC-P and tested in Mouse, Human samples. Cited in 30 publications.

Recombinant   KO Validated

Alternative names= YAP, YKI, COB1, YAP1, YAP2, YAP-1, YAP65, YAp 1, YAP 65, YAP1_HUMAN, Yorkie homolog, Protein yorkie homolog, Yes associated protein 1, Yes associated protein 2, Yes-associated protein 1, yes associated protein beta, 65 kDa Yes associated protein, 65 kDa Yes-associated protein, yes -associated protein delta, Yes associated protein 1 65kDa, Transcriptional coactivator YAP1, Yes-associated protein YAP65 homolog

Key facts

Host species	Rabbit
Form	Liquid
Isotype	IgG
Clonality	Monoclonal
Conjugation	Unconjugated
Clone number	EPR19812
Purification technique	Affinity purification Protein A
Specificity	ab205270 is specific to the active (non-phosphorylated) form of YAP1.

Reactivity data

WB

Tested

Species	Mouse
Dilution info	1/1000
Notes	-

Species	Human
Dilution info	1/1000
Notes	-

ICC/IF

Tested

Species	Human
Dilution info	1/500
Notes	-

Expected

Species	Mouse
Dilution info	Use at an assay dependent concentration.
Notes	-

IHC-P

Tested

Species	Mouse
Dilution info	1/2000
Notes	-

Species	Human
Dilution info	1/2000
Notes	-

Storage

Shipped at conditions	Blue Ice

Appropriate short term storage duration	1-2 weeks
Appropriate short term storage conditions	+4°C
Appropriate long term storage conditions	-20°C
Aliquoting information	Upon delivery aliquot
Storage information	Avoid freeze / thaw cycle

Notes

Our RabMAb® technology is a patented hybridoma-based technology for making rabbit monoclonal antibodies. For details on our patents, please refer to RabMAb® patents

This product is a recombinant monoclonal antibody, which offers several advantages including:

- High batch-to-batch consistency and reproducibility
- Improved sensitivity and specificity
- Long-term security of supply
- Animal-free batch production

Product promise

Tested

We have tested this species and application combination and it works. It is covered by our product promise.

Expected

We have not tested this specific species and application combination in-house, but expect it will work. It is covered by our product promise.

Predicted

This species and application combination has not been tested, but we predict it will work based on strong homology. However, this combination is not covered by our product promise.

Not recommended

We do not recommend this combination. It is not covered by our product promise.

SAV1 Monoclonal Antibody (OTI2B7)

**Product Details**	
Size	100 µL
Species Reactivity	Human
Host/Isotype	Mouse / IgG1
Class	Monoclonal
Type	Antibody
Clone	OTI2B7
Conjugate	Unconjugated
Immunogen	Human recombinant protein fragment corresponding to amino acids 167-383 of SAV1 produced in E.coli
Form	Liquid
Concentration	1 mg/mL
Purification	Affinity Chromatography
Storage buffer	PBS, pH 7.3, with 1% BSA, 50% glycerol
Contains	0.02% sodium azide
Storage conditions	-20° C, Avoid Freeze/Thaw Cycles
RRID	AB_2725297

**Applications**	**Tested Dilution**	**Publications**
Western Blot (WB)	1:2,000	-
Immunohistochemistry (Paraffin) (IHC (P))	1:150	-

Product Images For SAV1 Monoclonal Antibody (OTI2B7)

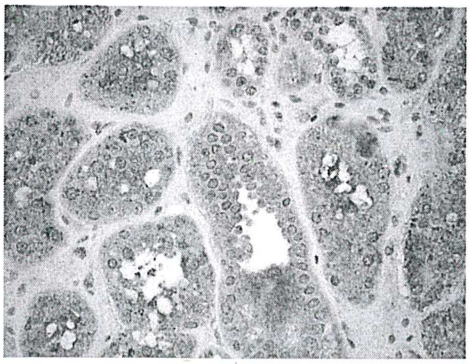

SAV1 Antibody (MA5-26689) in IHC (P)
Immunohistochemistry was performed on paraffin-embedded human kidney tissue. To expose target proteins, 1 mM EDTA in 10mM Tris, pH8.0 was used. Following antigen retrieval, tissues were probed with a SAV1 monoclonal antibody (Product # MA5-26689) at a dilution of 1:150.

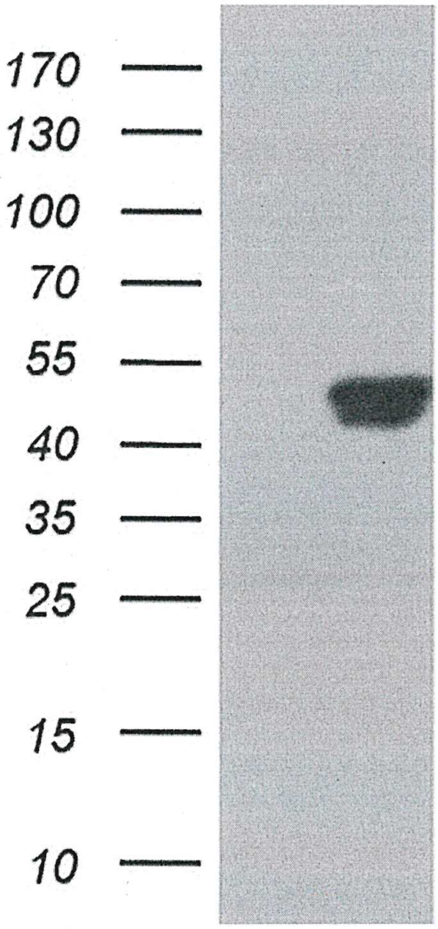

SAV1 Antibody (MA5-26689) in WB
Western blot analysis of SAV1 in HEK293T cells in untransfected (Left lane) and transfected (Right lane) samples using 5 µg per lane. The samples were separated by SDS-PAGE and probed with SAV1 (Product # MA5-26689) monoclonal antibody.

For Research Use Only. Not for use in diagnostic procedures. Not for resale without express authorization. Products are warranted to operate or perform substantially in conformance with published Product specifications in effect at the time of sale, as set forth in the Production documentation, specifications and/or accompanying package inserts ("Documentation"). No claim of suitability for use in applications regulated by FDA is made. The warranty provided herein is valid only when used by properly trained individuals. Unless otherwise stated in the Documentation, this warranty is limited to one year from date of shipment when the Product is subjected to normal, proper and intended usage. This warranty does not extend to anyone other than the Buyer. Any model or sample furnished to Buyer is merely illustrative of the general type and quality of goods and does not represent that any Product will conform to such model or sample. NO OTHER WARRANTIES, EXPRESS OR IMPLED, ARE GRANTED INCLUDING WITHOUT LIMITATION, IMPLIED WARRANTIES OF MERCHANTABILITY, FITNESS FOR ANY PARTICULAR PURPOSE, OR NON INFRINGEMENT. BUYER'S EXCLUSIVE REMEDY FOR NON-CONFORMING PRODUCTS DURING THE WARRANTY PERIOD IS LIMITED TO REPAIR, REPLACEMENT OF OR REFUND FOR THE NON-CONFORMING PRODUCT(S) AT SELLER'S SOLE OPTION. THERE IS NO OBLIGATION TO REPAIR, REPLACE OR REFUND FOR PRODUCTS AS THE RESULT OF (I) ACCIDENT, DISASTER OR EVENT OF FORCE MAJEURE, (II) MISUSE, FAULT OR NEGLIGENCE OF OR BY BUYER, (III) USE OF THE PRODUCTS IN A MANNER FOR WHICH THEY WERE NOT DESIGNED, OR (IV) IMPROPER STORAGE AND HANDLING OF THE PRODUCTS. Unless otherwise expressly stated on the Product or in the documentation accompanying the Product, the Product is intended for research only and is not to be used for any other purpose, including without limitation, unauthorized commercial uses, in vitro diagnostic uses, ex vivo or in vivo therapeutic uses, or any type of consumption by or application to human or animals.

YAP1 Monoclonal Antibody (1A12)

Product Details	
Size	100 µg
Species Reactivity	Human
Host/Isotype	Mouse / IgG1
Class	Monoclonal
Type	Antibody
Clone	1A12
Conjugate	Unconjugated
Immunogen	Purified recombinant fragment of human YAP1 expressed in E. Coli.
Form	Liquid
Concentration	1 mg/mL
Purification	Protein G
Storage buffer	PBS
Contains	0.05% sodium azide
Storage conditions	Store at 4°C short term. For long term storage, store at -20°C, avoiding freeze/thaw cycles.
RRID	AB_2538671

Applications	Tested Dilution	Publications
Western Blot (WB)	1:500-1:2,000	-
Immunohistochemistry (Paraffin) (IHC (P))	1:200-1:1,000	-
Flow Cytometry (Flow)	1:200-1:400	-
ELISA (ELISA)	1:10,000	-

Product Specific Information

MA5-17200 targets YAP1 in indirect ELISA, FACS, IHC, and WB applications and shows reactivity with Human samples.

The MA5-17200 immunogen is purified recombinant fragment of human YAP1 expressed in E. Coli.

Product Images For YAP1 Monoclonal Antibody (1A12)

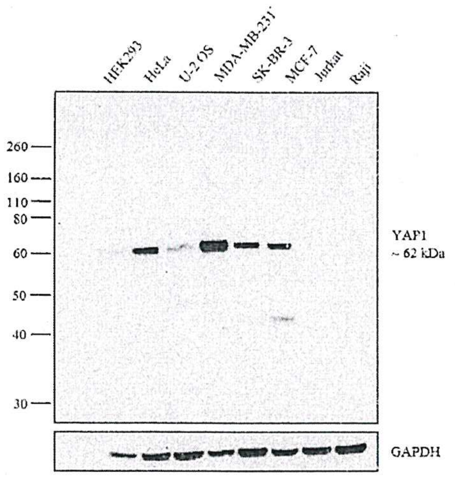

YAP1 Antibody (MA5-17200)
Antibody specificity was demonstrated by detection of differential basal expression of the target across cell models owing to their inherent genetic constitution. Relative expression of YAP1 was observed in differentiated somatic cell lines (HEK293, HeLa, U-2 OS, MDA-MB-231, SK-BR-3 and MCF-7) in comparison to lymphoid cell lines (Jurkat and Raji) using YAP1 antibody (Product # MA5-17200) in western blot. {RE}

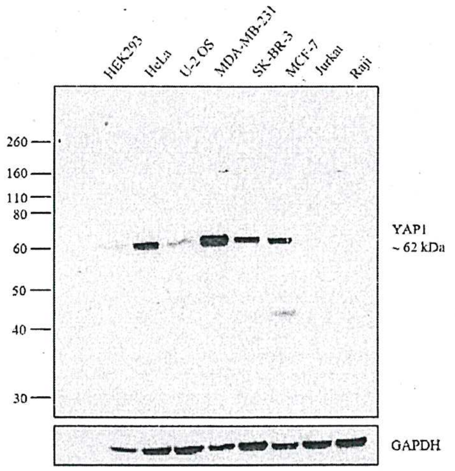

YAP1 Antibody (MA5-17200) in WB
Western blot analysis was performed on whole cell extracts (30 µg lysate) of HEK293 (Lane 1), HeLa (Lane 2), U-2 OS (Lane 3), MDA-MB-231 (Lane 4), SK-BR-3 (Lane 5), MCF-7 (Lane 6), Jurkat (Lane 7) and Raji (Lane 8). The blot was probed with Anti-YAP1 Monoclonal Antibody (Product # MA5-17200, 1:2000 dilution) and detected by chemiluminescence using Goat anti-Mouse IgG (H+L) Superclonal™ Secondary Antibody, HRP conjugate (Product # A28177, 0.25 µg/ml, 1:4000 dilution). A 62 kDa band corresponding to YAP1 was observed across all the cell lines tested except Jurkat and Raji.

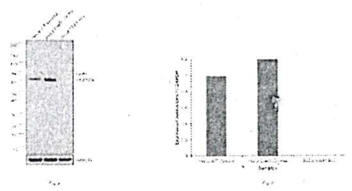

YAP1 Antibody (MA5-17200)
Antibody specificity was demonstrated by CRISPR-Cas9 mediated knockout of target protein. A loss of signal was observed for target protein inYAP1 KO cell line compared to control cell line using Anti-YAP1 Monoclonal Antibody (1A12) (Product # MA5-17200). {KO}

View more figures on thermofisher.com

For Research Use Only

WWTR1 Monoclonal antibody

TAZ

Catalog Number:66500-1-Ig Featured Product 8 Publications

proteintech®
Antibodies | ELISA kits | Proteins
www.ptglab.com

Basic Information

Catalog Number:
66500-1-Ig

Size:
150ul , Concentration: 2522 μg/ml by Nanodrop and 1000 μg/ml by Bradford method using BSA as the standard;

Source:
Mouse

Isotype:
IgG1

Immunogen Catalog Number:
AG13330

GenBank Accession Number:
BC014052

GeneID (NCBI):
25937

Full Name:
WW domain containing transcription regulator 1

Calculated MW:
44 kDa

Observed MW:
55 kDa

Purification Method:
Protein A purification

CloneNo.:
2A12A10

Recommended Dilutions:
WB 1:5000-1:50000
IHC 1:300-1:1200
IF 1:200-1:800

Applications

Tested Applications:
IF, IHC, WB, ELISA

Cited Applications:
IF, IHC, WB

Species Specificity:
Human, rat, mouse

Cited Species:
human, rat, mouse

Note-IHC: suggested antigen retrieval with TE buffer pH 9.0; (*) Alternatively, antigen retrieval may be performed with citrate buffer pH 6.0

Positive Controls:

WB : A549 cells, HepG2 cells, HSC-T6 cells, MCF-7 cells, NIH/3T3 cells, MDA-MB-231 cells, HeLa cells, Jurkat cells, 4T1 cells, MDA-MB-453S cells

IHC : human liver cancer tissue, human breast cancer tissue

IF : human liver cancer tissue,

Background Information

TAZ, also referred as WWTR1, is a transcriptional co-activator with a PDZ-binding motif that is regulated by its interaction with 14-3-3 protein. TAZ is expressed in many primary tumors, such as breast cancer, thyroid carcinoma, colorectal cancer, and glioma. TAZ has been reported to be one of the nuclear effectors of Hippo-related pathways that regulate organ size control, cancer stem cell (CSC) properties, and epithelial-mesenchymal transition (EMT). TAZ has been defined for its role in the nucleus, where it functions directly as a transcriptional regulator by interacting with several nuclear factors as Runx2/Cbfa1, NHERF-2, TEF-1, TBX5, PAX3, PAX8 and TTF-1.

Notable Publications

Author	Pubmed ID	Journal	Application
Xinghe Chen	36414118	Toxicol Appl Pharmacol	WB,IF
Xuyang Hu	35833021	Front Pharmacol	WB,IHC,IF
Adheesh Bhandari	31312369	Am J Transl Res	WB

Storage

Storage:
Store at -20°C. Stable for one year after shipment.
Storage Buffer:
PBS with 0.02% sodium azide and 50% glycerol pH 7.3.
Aliquoting is unnecessary for -20°C storage

*** 20ul sizes contain 0.1% BSA

Selected Validation Data

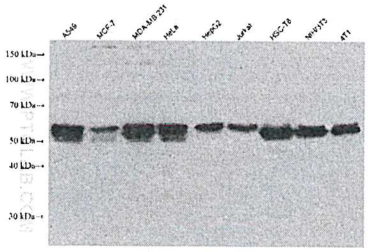

Various lysates were subjected to SDS PAGE followed by western blot with 66500-1-Ig (TAZ antibody) at dilution of 1:10000 incubated at room temperature for 1.5 hours.

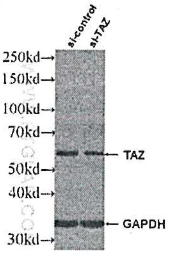

WB result of TAZ antibody (66500-1-Ig; 1:8000; incubated at room temperature for 1.5 hours) with sh-Control and sh-TAZ transfected HepG2 cells.

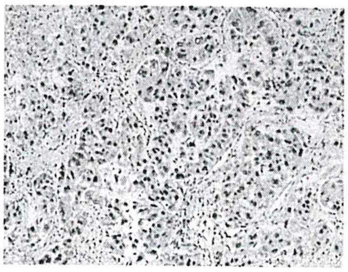

Immunohistochemical analysis of paraffin-embedded human liver cancer tissue slide using 66500-1-Ig (TAZ antibody) at dilution of 1:600 (under 10x lens. Heat mediated antigen retrieval with Tris-EDTA buffer (pH 9.0).

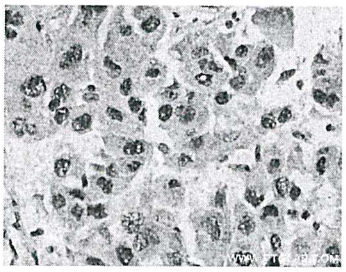

Immunohistochemical analysis of paraffin-embedded human liver cancer tissue slide using 66500-1-Ig (TAZ antibody) at dilution of 1:600 (under 40x lens. Heat mediated antigen retrieval with Tris-EDTA buffer (pH 9.0).

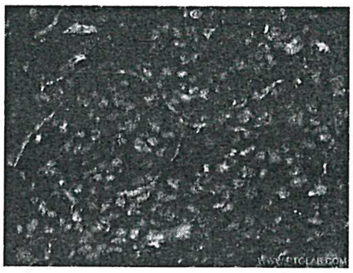

Immunofluorescent analysis of (4% PFA) fixed human liver cancer tissue using TAZ antibody (66500-1-Ig, Clone: 2A12A10) at dilution of 1:400 and CoraLite®488-Conjugated AffiniPure Goat Anti-Mouse IgG(H+L).

invitrogen

Catalog # PA5-22015

MST1 (STK4) Polyclonal Antibody

Product Details	
Size	100 µL
Species Reactivity	Human, Mouse
Host/Isotype	Rabbit / IgG
Class	Polyclonal
Type	Antibody
Conjugate	Unconjugated
Immunogen	Synthetic peptide corresponding to a region within amino acids 324 and 418 of STK4 (Uniprot ID#Q13043)
Form	Liquid
Concentration	1 mg/mL
Purification	Antigen affinity chromatography
Storage buffer	0.1M tris glycine, pH 7, with 10% glycerol
Contains	0.01% thimerosal
Storage conditions	Store at 4°C short term. For long term storage, store at -20°C, avoiding freeze/thaw cycles.
RRID	AB_11157025

Applications	Tested Dilution	Publications
Western Blot (WB)	1:500-1:3,000	2 Publications
Immunohistochemistry (Paraffin) (IHC (P))	1:100-1:1,000	-
Immunocytochemistry (ICC/IF)	1:100-1:1,000	-
Immunoprecipitation (IP)	1:100-1:500	-

Product Specific Information

Recommended positive controls: HepG2, mouse brain.

Predicted reactivity: Mouse (100%), Rat (100%), Chicken (100%), Rhesus Monkey (100%), Chimpanzee (100%), Bovine (100%).

Store product as a concentrated solution. Centrifuge briefly prior to opening the vial.

Product Images For MST1 (STK4) Polyclonal Antibody

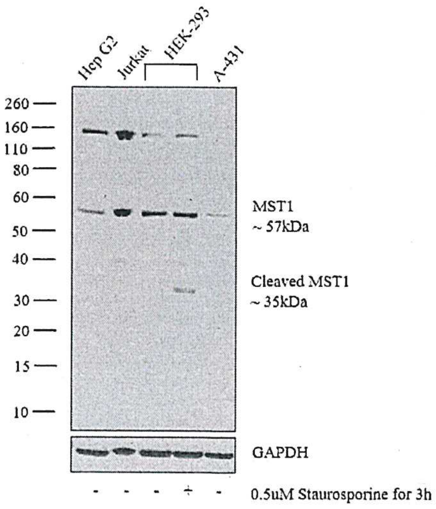

MST1 (STK4) Antibody (PA5-22015) in WB

Western blot analysis was performed on whole cell extracts (30 µg lysate) of Hep G2 (Lane 1), Jurkat (Lane 2), HEK-293 (Lane 3), HEK-293 treated with Staurosporine (0.5uM for 3h) (Lane 4) and A-431 (Lane 5). The blot was probed with Anti-MST1 Polyclonal Antibody (Product # PA5-22015, 1:1000 dilution) and detected by chemiluminescence using Goat anti-Rabbit IgG (Heavy Chain) Superclonal™ Secondary Antibody, HRP conjugate (Product # A27036, 0.25 µg/mL, 1:4000 dilution). A 57kDa band corresponding to MST1 was observed across all the cell lines tested. A cleaved fragment was observed to be induced upon treatment of HEK-293 cells with Staurosporine. An uncharacterized band was observed at 160kDa.

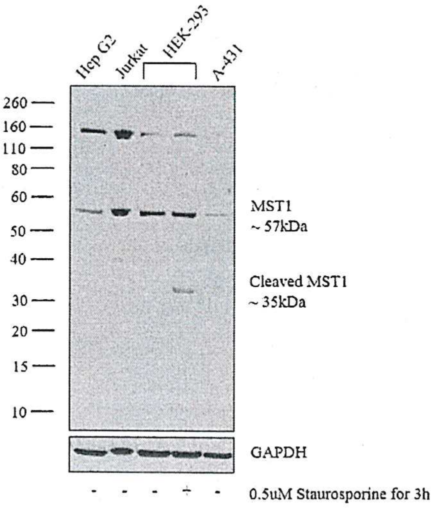

MST1 (STK4) Antibody (PA5-22015)

Altered expression of target protein upon cell treatment demonstrates antibody specificity. Western blot analysis of MST1 using with Anti-MST1 Polyclonal Antibody (Product # PA5-22015) shows cleavage of the MST1 upon treatment of HEK-293 cells with Staurosporine. {TM}

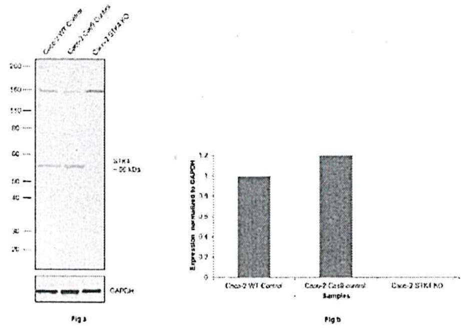

MST1 (STK4) Antibody (PA5-22015)

Antibody specificity was demonstrated by CRISPR-Cas9 mediated knockout of target protein. A loss of signal was observed for target protein inSTK4 KO cell line compared to control cell line using a MST1 (STK4) Polyclonal Antibody (Product # PA5-22015). {KO}

TEAD4 Polyclonal Antibody

Product Details	
Size	100 µL
Species Reactivity	Human
Host/Isotype	Rabbit / IgG
Class	Polyclonal
Type	Antibody
Conjugate	Unconjugated
Immunogen	Recombinant fragment corresponding to a region within amino acids 1 and 260 of TEAD4 (Uniprot ID#Q15561)
Form	Liquid
Concentration	1.1 mg/mL
Purification	Antigen affinity chromatography
Storage buffer	PBS, pH 7, with 20% glycerol
Contains	0.025% ProClin 300
Storage conditions	Store at 4°C short term. For long term storage, store at -20°C, avoiding freeze/thaw cycles.
RRID	AB_11153439

Applications	Tested Dilution	Publications
Western Blot (WB)	1:500-1:3,000	-
Immunohistochemistry (Paraffin) (IHC (P))	1:100-1:1,000	-
Immunocytochemistry (ICC/IF)	1:100-1:1,000	-

Product Specific Information

Recommended positive controls: HepG2, HepG2 nuclear extract.

Predicted reactivity: Mouse (91%), Pig (94%), Chicken (85%).

Store product as a concentrated solution. Centrifuge briefly prior to opening the vial.
