# Supplementary material for: Hippo Pathway Dysregulation in Thymic Epithelial Tumors (TETs): Associations with Clinicopathological Features and Patients’ Prognosis
Source: Int J Mol Sci. 2025 Jun 20;26(13):5938. doi: 10.3390/ijms26135938 (PMC12250049; doi:10.3390/ijms26135938)
Supplement: Supplementary file 1 [file ijms-26-05938-s001.zip › S2 Validation Protocols Antibodies IHC.pdf]

(1)

Klinikum Nürnberg

Institut für Pathologie<br>Klinikum Nürnberg	**Antikörper-Austestung**<br>**Formblatt**	Dok.-Nr.: 350<br>Version: 1.0

(Änderung: Ab Version 2.0)

Antikörper: LATS1 **Klon:** polyclonal **Firma:** proteintech

Artikelnummer: 17049-1-AP **Charge:** 00108706 **Verfallsdatum:** /

Fall #, Testgewebe: 1.) H20806/17 I7 **2.)** H49567/19 I3 **3.)** ____ **4.)** ____

OT-Nr.	Fall / Testgewebe	Vorbehandlung TR 9,0	TR 6,1	Sonstige	Verdünnung	Inkubationszeit Primärantikörper	Nachweissystem	Linker M	R	Vorlagedatum	Kürzel MTA	Bewertung	Kürzel Arzt
1	1	X			1:200	30	Envision Flex			14.03.24	Le	+++ mit Hintergrund?	Lg
2	1		X		1:200	30	"			"	Le	- Ø Färbung	Lg
3	1	X			1:200	30	"		X	"	Le	+++ Viel Hintergrund	Lg
4	~~1~~ 2	X			1:250	30	"			19.03.24	Le		Lg
5	1	X			1:250	20	"			19.03.24	Le	+ schwache F.	Lg
6	1	X			1:200	20	"			19.03.24	Le	+ schwache F.	Lg
7	1	X			1:250	30	"			26.03.24	Le	+++ Sehr starke Färbung X — Falsche Färbung	Lg
8	1	X			1:300	30	"			28.03.24	Le	+/- Sehr schwach	Lg
9	1	X			1:350	30	"			28.03.24	Le	+/- sehr schwach	Lg

TR: Target Retrieval 9,0: pH 9,0 / 6,1: pH 6,1 M: Mouse-Linker R: Rabbit-Linker

Auswertung:

Beste Ergebnisse mit den Parametern aus Test (OT)-<br>Nr. 1	Für anschließende Validierung zu verwendende Fallnummern / Gewebe:	Bemerkungen:<br>Forschung → keine Validierung nötig	Datum, Unterschrift Facharzt

Die Archivierung dieser Aufzeichnungen erfolgt in der jeweiligen Abteilung für 10 Jahre.

(2)

Klinikum Nürnberg
wir sind für Sie da.

Institut für Pathologie<br>Klinikum Nürnberg	**Antikörper-Austestung**<br>**Formblatt**	Dok.-Nr.: 350<br>Version: 1.0

(Änderung: Ab Version 2.0)

Antikörper: LATS1 **Klon:** polyclonal **Firma:** proteintech

Artikelnummer: 17049-1-AP **Charge:** 00108706 **Verfallsdatum:** /

Fall #, Testgewebe: 1.) H2080617I7 **2.)** ____ **3.)** ____ **4.)** ____

OT-Nr.	Fall / Testgewebe	Vorbehandlung TR 9,0	Vorbehandlung TR 6,1	Vorbehandlung Sonstige	Verdünnung	Inkubationszeit Primärantikörper	Nachweissystem	Linker M	Linker R	Vorlagedatum	Kürzel MTA	Bewertung	Kürzel Arzt
10	1	X			1:300	40'	Envision Flex			28.03.24	ke	++ leicht schwach	lg
11	1	X			1:250	25'	"			"	ke	+ eher schwach	lg
12	1	X			1:350	20'	"		X	"	ke	+ eher schwach	lg
13	1	X			1:250	30'	"			03.04.24	ke	++ schwache f	lg.
14	1	X			1:200	25'	"			03.04.24	ke	++ schwache f.	lg.

TR: Target Retrieval 9,0: pH 9,0 / 6,1: pH 6,1 M: Mouse-Linker R: Rabbit-Linker

Auswertung:

Beste Ergebnisse mit den Parametern aus Test (OT)-Nr. 1	Für anschließende Validierung zu verwendende Fallnummern / Gewebe:	Bemerkungen: Forschung → keine Validierung notwendig	12.4.24 [signature]<br>Datum, Unterschrift Facharzt

Die Archivierung dieser Aufzeichnungen erfolgt in der jeweiligen Abteilung für 10 Jahre.

Klinikum Nürnberg
wir sind für Sie da.

Institut für Pathologie<br>Klinikum Nürnberg	**Antikörper-Austestung**<br>**Formblatt**	Dok.-Nr.: 350<br>Version: 1.0

(Änderung: Ab Version 2.0)

Antikörper: MOB1 **Klon:** Polyclonal **Firma:** invitrogen

Artikelnummer: PAS-98902 **Charge:** ZB4235178A **Verfallsdatum:** ______

Fall #, Testgewebe: 1.) H20806117-I7 **2.)** ______ **3.)** ______ **4.)** ______

OT-Nr.	Fall / Testgewebe	Vorbehandlung TR 9,0	TR 6,1	Sonstige	Verdünnung	Inkubationszeit Primärantikörper	Nachweissystem	Linker M	R	Vorlagedatum	Kürzel MTA	Bewertung	Kürzel Arzt
1	~~H208061~~ 1	X			1:1000	30	Envision Flex			19.03.24	ke	–	Lg
2	1		X		1:1000	"	"			19.03.24	ke	–	Lg
3	1	X			1:1000	"	"		X	19.03.24	ke	– Fragliche Flare unspezifische Reaktion	Lg
4	1	X			1:500	"	"			26.03.24	ke	–/+	Lg
5	1		X		1:500	"	"			26.03.24	ke	–/+	Lg
6	1	X			1:500	"	"		X	26.03.24	ke	+/–	Lg
7	1	X			1:350	~~1~~ 40	"		X	28.03.24	ke	+++ ohne Hintergrund	Lg
8	1	X			1:250	~~1~~ 40	"		X	28.03.24	ke	+++ " –	Lg

TR: Target Retrieval 9,0: pH 9,0 / 6,1: pH 6,1 M: Mouse-Linker R: Rabbit-Linker

Auswertung:

Beste Ergebnisse mit den Parametern aus Test (OT)- Nr. 7	Für anschließende Validierung zu verwendende Fallnummern / Gewebe:	Bemerkungen: Forschung → keine Validierung nötig	28.3.24 [signature]<br>Datum, Unterschrift Facharzt

Die Archivierung dieser Aufzeichnungen erfolgt in der jeweiligen Abteilung für 10 Jahre.

Institut für Pathologie<br>Klinikum Nürnberg	**Antikörper-Austestung**<br>**Formblatt**	Dok.-Nr.: 350<br>Version: 1.0

(Änderung: Ab Version 2.0) (interne Bez.: AYAP)

Antikörper: Anti-active YAP1 **Klon:** EPR19812 **Firma:** abcam

Artikelnummer: ab205270 **Charge:** 1052726-8 **Verfallsdatum:** /

Fall #, Testgewebe: 1.) H20806117 I7 **2.)** ___ **3.)** ___ **4.)** ___

OT-Nr.	Fall / Testgewebe	Vorbehandlung			Verdünnung	Inkubationszeit Primärantikörper	Nachweissystem	Linker		Vorlagedatum	Kürzel MTA	Bewertung	Kürzel Arzt
		TR 9,0	TR 6,1	Sonstige				M	R				
1	1	X			1:2000	30 Min.	Envision Flex			14.03.24	[illegible]	+++ viel Zytoplasmatische f.	[illegible]
2	1		X		1:2000	30 Min	"			14.03.24	[illegible]	klarer Färbung: +++, [illegible] nukleär	[illegible]
3	1	X			1:2000	30 Min.	"		X	14.03.24	[illegible]	viel Hintergrund	[illegible]

TR: Target Retrieval 9,0: pH 9,0 / 6,1: pH 6,1 M: Mouse-Linker R: Rabbit-Linker

Auswertung:

Beste Ergebnisse mit den Parametern aus Test (OT)- Nr. 1 2	Für anschließende Validierung zu verwendende Fallnummern / Gewebe:	Bemerkungen: Forschung → keine Validierung nötig	28.3.24 [signature]<br>Datum, Unterschrift Facharzt

Die Archivierung dieser Aufzeichnungen erfolgt in der jeweiligen Abteilung für 10 Jahre.

Institut für Pathologie<br>Klinikum Nürnberg	**Antikörper-Austestung**<br>**Formblatt**	Dok.-Nr.: 350<br>Version: 1.0

(Änderung: Ab Version 2.0)

Antikörper: SAV1 **Klon:** OT12B7 **Firma:** invitrogen

Artikelnummer: MA5-26689 **Charge:** ZB4235305 **Verfallsdatum:** ____

Fall #, Testgewebe: 1.) H4443122 10 **2.)** ____ **3.)** ____ **4.)** ____

OT-Nr.	Fall / Testgewebe	Vorbehandlung TR 9,0	Vorbehandlung TR 6,1	Vorbehandlung Sonstige	Verdünnung	Inkubationszeit Primärantikörper	Nachweissystem	Linker M	Linker R	Vorlagedatum	Kürzel MTA	Bewertung	Kürzel Arzt
1	1	X			1:~~1~~150	30	Envision Flex			19.3.24	k	-/+ schwache granuläre F. bei einzelnen Tubuli	Lg
2	1		X		1:150	30	"			19.3.24	k	-/+ "	Lg
3	1	X			1:150	30	"	X		19.3.24	k	-/+ "	Lg
4	1				1:150	~~720~~ 540	"	X		26.03.24	k	++ zytoplasmatisch	Lg
5	1	X			1:100	~~720~~ 540	"	X		26.03.24	k	+++ zytoplasmatisch	Lg

TR: Target Retrieval 9,0: pH 9,0 / 6,1: pH 6,1 M: Mouse-Linker R: Rabbit-Linker

Auswertung:

Beste Ergebnisse mit den Parametern aus Test (OT)-Nr.	Für anschließende Validierung zu verwendende Fallnummern / Gewebe:	Bemerkungen:	Datum, Unterschrift Facharzt
5		Forschung → keine Validierung notwendig	26.3.24 [signature]

Die Archivierung dieser Aufzeichnungen erfolgt in der jeweiligen Abteilung für 10 Jahre.

Institut für Pathologie<br>Klinikum Nürnberg	**Antikörper-Austestung**<br>**Formblatt**	Dok.-Nr.: 350<br>Version: 1.0

(Änderung: Ab Version 2.0)

Antikörper: YAP1 **Klon:** 1A12 **Firma:** invitrogen

Artikelnummer: MA5-17200 **Charge:** VA2914967 **Verfallsdatum:** /

Fall #, Testgewebe: 1.) H6035124 I8 **2.)** ____ **3.)** ____ **4.)** ____

OT-Nr.	Fall / Testgewebe	Vorbehandlung TR 9,0	Vorbehandlung TR 6,1	Vorbehandlung Sonstige	Verdünnung	Inkubationszeit Primärantikörper	Nachweissystem	Linker M	Linker R	Vorlagedatum	Kürzel MTA	Bewertung	Kürzel Arzt
1	1	X			1:1000	30	Envision Flex			14.03.24	[illegible]	+++ ohne Hintergrund	Lg
2	1		X		1:1000	30	"			"	[illegible]	++ sehr schwache Färbung.	Lg
3	1	X			1:1000	30	"	X		"	[illegible]	viel Hintergrund +++	Lg

TR: Target Retrieval 9,0: pH 9,0 / 6,1: pH 6,1 M: Mouse-Linker R: Rabbit-Linker

Auswertung:

Beste Ergebnisse mit den Parametern aus Test (OT)- Nr. 1	Für anschließende Validierung zu verwendende Fallnummern / Gewebe:	Bemerkungen: Forschung -> keine Validierung nötig	28.3.24 [Unterschrift]<br>Datum, Unterschrift Facharzt

Die Archivierung dieser Aufzeichnungen erfolgt in der jeweiligen Abteilung für 10 Jahre.

Institut für Pathologie<br>Klinikum Nürnberg	**Antikörper-Austestung**<br>**Formblatt**	Dok.-Nr.: 350<br>Version: 1.0

(Änderung: Ab Version 2.0)

Antikörper: TAZ **Klon:** 2A12A10 **Firma:** proteintech

Artikelnummer: 66500-1-Ig **Charge:** 10021426 **Verfallsdatum:** /

Fall #, Testgewebe: 1.) H20806117 I7 **2.)** ____ **3.)** ____ **4.)** ____

OT-Nr.	Fall / Testgewebe	Vorbehandlung			Verdünnung	Inkubationszeit Primärantikörper	Nachweissystem	Linker		Vorlagedatum	Kürzel MTA	Bewertung	Kürzel Arzt
		TR 9,0	TR 6,1	Sonstige				M	R				
1	1	X			1:600	30	Envision Flex				Le	-/+ einzelne Positivitäten	[illegible]
2	1		X		1:600	30	"				Le	- Ø Färbung	[illegible]
3	1	X			1:600	30	"	X			Le	++ nukl + zytoplasmatisch	[illegible]

TR: Target Retrieval 9,0: pH 9,0 / 6,1: pH 6,1 M: Mouse-Linker R: Rabbit-Linker

Auswertung:

Beste Ergebnisse mit den Parametern aus Test (OT)- Nr. 3	Für anschließende Validierung zu verwendende Fallnummern / Gewebe:	Bemerkungen: Forschung -> keine Validierung nötig	28.3.24<br>Datum, Unterschrift Facharzt

Die Archivierung dieser Aufzeichnungen erfolgt in der jeweiligen Abteilung für 10 Jahre.

Institut für Pathologie<br>Klinikum Nürnberg	**Antikörper-Austestung**<br>**Formblatt**	Dok.-Nr.: 350<br>Version: 1.0

(Änderung: Ab Version 2.0)

Antikörper: MST1 **Klon:** polyclonal **Firma:** invitrogen

Artikelnummer: PA5-22015 **Charge:** ZB4234885B **Verfallsdatum:** /

Fall #, Testgewebe: 1.) H49567/19 I3 **2.)** ______ **3.)** ______ **4.)** ______

OT-Nr.	Fall / Testgewebe	Vorbehandlung			Verdünnung	Inkubationszeit Primärantikörper	Nachweissystem	Linker		Vorlagedatum	Kürzel MTA	Bewertung	Kürzel Arzt
		TR 9,0	TR 6,1	Sonstige				M	R				
1	1	X			1:500	30	Envision Flex			14.3.24	Le	+/- sehr schwach zytoplasm.	Lg
2	1		X		1:500	30	"			14.3.24	Le	-/+ fast keine f.	Lg
3	1	X			1:500	30	"		X	14.3.24	Le	+ schwach jedoch eher spezifisch	Lg
4	1	X			1:250	30	"			19.3.24	Le	++ Makrophagen +/- Tumorzellen	Lg
5	1	X			1:250	40	"			19.3.24	Le	++ Makrophagen - Tumorzellen	Lg

TR: Target Retrieval 9,0: pH 9,0 / 6,1: pH 6,1 M: Mouse-Linker R: Rabbit-Linker

Auswertung:

Beste Ergebnisse mit den Parametern aus Test (OT)-<br>Nr. 4	Für anschließende Validierung zu verwendende Fallnummern / Gewebe:	Bemerkungen:<br>Forschung -> keine Validierung nötig	28.3.24 [signature]<br>Datum, Unterschrift Facharzt

Die Archivierung dieser Aufzeichnungen erfolgt in der jeweiligen Abteilung für 10 Jahre.

Institut für Pathologie<br>Klinikum Nürnberg	**Antikörper-Austestung**<br>**Formblatt**	Dok.-Nr.: 350<br>Version: 1.0

(Änderung: Ab Version 2.0)

Antikörper: TEAD4 **Klon:** Polyclonal **Firma:** invitrogen

Artikelnummer: PA5-21977 **Charge:** ZC4234883 **Verfallsdatum:** ____

Fall #, Testgewebe: 1.) HS541/24I3 **2.)** ____ **3.)** ____ **4.)** ____

OT-Nr.	Fall / Testgewebe	Vorbehandlung TR 9,0	Vorbehandlung TR 6,1	Vorbehandlung Sonstige	Verdünnung	Inkubationszeit Primärantikörper	Nachweissystem	Linker M	Linker R	Vorlagedatum	Kürzel MTA	Bewertung	Kürzel Arzt
1	1	X			1:500	30	Envision Flex			19.3.24	le	+++ positive Zellen nukleär & zytopl.	Gs
2	1		X		"	"	"			19.3.24	le	+++ leicht schwächer Färbung	Gs
3	1	X			"	"	"		X	19.3.24	le	+++ zu starke F.	Gs

TR: Target Retrieval 9,0: pH 9,0 / 6,1: pH 6,1 M: Mouse-Linker R: Rabbit-Linker

Auswertung:

Beste Ergebnisse mit den Parametern aus Test (OT)-<br>Nr. 1	Für anschließende Validierung zu verwendende Fallnummern / Gewebe:	Bemerkungen:<br>Forschung, keine Validierung nötig	28.3.24 [signature]<br>Datum, Unterschrift Facharzt

Die Archivierung dieser Aufzeichnungen erfolgt in der jeweiligen Abteilung für 10 Jahre.
